# Cardiac Rehabilitation and Cognitive Impairment: Elective Affinities or Fatal Attraction

**DOI:** 10.3390/jcm15124598

**Published:** 2026-06-13

**Authors:** Valeria Visco, Francesco Loria, Antonio Squillante, Francesca Palmieri, Federica Piani, Ilaria Fucile, Carmine Izzo, Maria Rosaria Rusciano, Cristina Gatto, Rónán O’Caoimh, David William Molloy, Costantino Mancusi, Giorgia Bruno, Nicola Virtuoso, Carmine Vecchione, Michele Ciccarelli

**Affiliations:** 1 Department of Medicine, Surgery, and Dentistry, University of Salerno, 84081 Salerno, Italy; vvisco@unisa.it (V.V.); crgatto@unisa.it (C.G.);; 2 Cardiac Rehabilitation Unit, University Hospital “San Giovanni di Dio e Ruggi d’Aragona”, 84131 Salerno, Italy; 3 Otto Loewi Research Center for Vascular Biology, Immunology and Inflammation, Division of Physiology and Pathophysiology, Medical University of Graz, 8010 Graz, Austria; 4 Department of Advanced Biomedical Sciences, “Federico II” University, 80131 Naples, Italy; 5 Cardiology Unit, University Hospital “San Giovanni di Dio e Ruggi d’Aragona”, 84131 Salerno, Italy; 6 Health Research Board Clinical Research Facility, University College Cork, Mercy University Hospital, T12WE28 Cork City, Ireland; 7 Vascular Physiopathology Unit, IRCCS Neuromed, 86077 Pozzilli, Italy

**Keywords:** cardiac rehabilitation, cognitive impairment, cognition, cognitive frailty, heart–brain axis, cardiovascular disease, coronary artery disease, heart failure, cardiac surgery

## Abstract

Cardiac rehabilitation (CR) is strongly recommended in secondary cardiovascular prevention; indeed, in patients after cardiac surgery or with coronary artery disease or heart failure, this intervention is recommended to decrease mortality, morbidity, and disability, and to improve quality of life and cardiorespiratory fitness. Moreover, each step of the cardiovascular continuum denotes a potential risk factor for the progression of cognitive frailty; this interaction is highly prevalent, affecting approximately one-third of all patients in cardiology settings. For these reasons, CR should consider the patient’s cognitive domain; however, cognitive assessment is still rarely integrated into standard CR protocols. Therefore, this comprehensive review presents current evidence and recent updates on the interaction between CR and cognitive impairment, focusing on physiological mechanisms, core components, benefits, and strategies for implementing CR in patients with cognitive frailty to optimize recovery and prognosis.

## 1. Introduction: The Heart-Brain Continuum

The heart–brain axis reflects bidirectional interactions between the cardiovascular and nervous systems, where autonomic and central neural mechanisms link neurological disorders to cardiac complications and, conversely, cardiac diseases to cerebrovascular events and cognitive impairment [1]. Each step of the cardiovascular continuum denotes a potential risk factor for the progression of cognitive frailty and, consequently, if the risk factor is not eliminated, for the progression of dementia [2]. This interaction is highly prevalent, affecting approximately one-third of all patients in cardiology settings [3]. In heart failure (HF) populations, cognitive impairment prevalence ranges from 14% to 81%, depending on the severity of the condition and the assessment tools utilized [4]. Following an acute myocardial infarction (MI), cognitive deficits are observed in 22% to 37% of patients [5]. Despite its significant impact on prognosis, cognitive impairment remains critically underdiagnosed in 50% to 80% of cardiology cases [6].

Chronic cerebral hypoperfusion resulting from impairment of cardiac output serves as the primary driver of neurocognitive decline within this continuum [3]. Reduced cardiac output deprives metabolic-demand regions like the hippocampus and prefrontal cortex of oxygen, leading to neuronal atrophy [3]. This state of hypoxia disrupts the blood–brain barrier, facilitating the entry of pro-inflammatory cytokines that trigger chronic neuroinflammation [3]. Furthermore, these vascular disturbances are strongly correlated with white matter degeneration, which significantly slows information-processing speed and impairs executive control [7]. Hypoxia also disrupts mitochondrial activity and increases oxidative stress, further compounding neuronal injury [3].

Participation in structured, exercise-based cardiac rehabilitation (CR) has emerged as a potent intervention to stabilize or improve cognitive outcomes in this vulnerable population [5]. Rehabilitation programs provide direct neurobiological benefits by improving vascular compliance and increasing cerebral blood flow (CBF) [7]. Clinical data show that improvements in cardiorespiratory fitness, measured as peak Metabolic Equivalent of Task (METs), are positively correlated with significant gains in psychomotor speed and complex attention [7]. Notably, these cognitive enhancements appear to occur independently of changes in a patient’s psychological or depressive state, suggesting a direct physiological impact on brain health [7]. The upregulation of neurotrophic factors, such as brain-derived neurotrophic factor (BDNF), during exercise may contribute to facilitating neurogenesis and synaptic plasticity [5]. To manage this axis effectively, international guidelines now mandate routine cognitive screening as part of the core components of patient assessment in CR [8]. The use of sensitive screening tools, such as the Montreal Cognitive Assessment (MoCA), is recommended over traditional tests to identify early executive deficits [8]. Moreover, the Quick Mild Cognitive Impairment (Qmci) test, a precise, sensitive, and specific screening method for mild cognitive impairment (MCI), measures spatial and temporal orientation, immediate and delayed recall, clock drawing, logical memory, and verbal fluency in just five minutes, making it very practical in clinical practice [9]. Remote combined interventions that integrate sequential exercise and cognitive training offer a comprehensive approach to maintaining independence in cardiac patients [10]. Such multidomain strategies are essential for reducing the high risks of rehospitalization and mortality associated with cognitive vulnerability [4]. Ultimately, preserving brain health must be considered a priority as critical as restoring cardiac function to ensure the long-term quality of life (QOL) and survival of people living with cardiovascular diseases (CVDs) [11].

Precisely, in this comprehensive narrative review, we synthesize current evidence and recent updates on CR and cognitive impairment, focusing on physiological mechanisms, core components, benefits, and strategies for implementing CR in patients with cognitive frailty to optimize recovery and prognosis.

## 2. Search Strategy

We performed a detailed search on cognitive impairment in three different cardiology areas: coronary artery disease (CAD), HF, and cardiac surgery. A literature search was conducted in PubMed, Scopus, Web of Science, and Google Scholar. The search terms included: cardiac rehabilitation; cognitive impairment; cognition; cognitive frailty; heart–brain axis; cardiovascular disease; coronary artery disease; heart failure; and cardiac surgery. The final search was completed in February 2026. No publication-date limits were applied in an effort to consider as many studies as possible. Moreover, both qualitative and quantitative reports were considered. The reviewers screened titles and abstracts, while full texts were reviewed for significant articles. Moreover, the reviewers screened the reference lists of included trials and meta-analyses for relevant articles. Non-English publications, pediatric case reports, and meeting abstracts without enough data were excluded.

## 3. Cognitive Impairment in Coronary Artery Disease

Clinical and epidemiological evidence has shown a link between CAD and cognitive impairment; in particular, the presence of CAD is associated with a 26% increased risk of dementia [12]. These two diseases share common risk factors such as arterial hypertension, smoking, and diabetes. Endothelial dysfunction, inflammation, and oxidative stress contribute to CVDs by inducing atherosclerosis, vascular damage, and impaired nitric oxide signaling, thereby promoting amyloidogenesis and tau hyperphosphorylation, which are common determinants of cognitive impairment [13]. The higher the number of cardiovascular risk factors, the greater the brain atrophy, the lower the gray matter volume, and the poorer the white matter health [14]. In addition, it is emerging that microvascular dysfunction in the brain and the heart is part of a particular pathological pathway affecting microcirculation. In people with CAD, the presence of coronary microcirculatory dysfunction, as determined by low coronary flow reserve (CFR), is linked to cerebral small vessel disease (CSVD), abnormal cerebral flow hemodynamics, and subtle but important cognitive impairment [15]. This is particularly evident in patients who underwent percutaneous coronary intervention (PCI) or coronary artery bypass grafting (CABG) and who had greater cognitive decline than age and education-matched patients with no clinical evidence of CAD [16]. Atherosclerosis may lead to cerebral hypoperfusion and hypoxia, resulting in neuronal destabilization and contributing to neurodegenerative processes [17].

Epidemiological studies have demonstrated a significant association between incident CAD and accelerated cognitive decline. Xie et al. followed patients with a prior cardiovascular event for 12 years and demonstrated that, following CAD diagnosis, global cognition, verbal memory, and temporal orientation scores deteriorated significantly faster than before the event [18]. Patients with a younger age at the onset of CAD had an increased risk of dementia [19]. A recent study by Samani et al. investigated how CAD impacts cerebral vascular and metabolic health and how these modifications are linked to cognitive function in multiple domains using quantitative magnetic resonance imaging (MRI) [20]. Precisely, CAD patients had widespread vascular and metabolic impairments, particularly with reduced cerebral blood flow, cerebrovascular reactivity (a measure of vascular reserve), and cerebral metabolic rate of oxygen (CMRO_2_), and on the other hand, a higher oxygen extraction fraction (OEF), consistent with insufficient oxygen delivery [20]. Importantly, lower cerebrovascular reactivity was associated with lower performance in the executive function area of cognition, while higher OEF was associated with poorer working memory, emphasizing the roles of vascular reserve and oxygen consumption in cognition [20].

In conclusion, the complex interactions between systemic inflammation, cerebral hypoperfusion, and autonomic dysregulation caused by myocardial infarction determine the development of cognitive impairment [21].

## 4. Cognitive Impairment in Heart Failure

Cognitive impairment is a common condition in patients with HF, with incidences from 40% to 60%; precisely, this condition is associated with poor outcomes, such as more frequent hospital readmissions and higher mortality [22]. This condition commonly affects learning, executive function, working memory, attention, and psychomotor speed, while language and visuospatial abilities are less frequently involved. Precisely, HF-related alterations in cerebral perfusion, such as vascular dysfunction, reduced cardiac output, and sympathovagal imbalance, may contribute to the increased vulnerability to cognitive impairment recorded in these patients [22]. Several imaging methods, including nuclear imaging, magnetic resonance imaging, and sonographic techniques, have been implemented to assess CBF in HF patients. More recent studies, however, have highlighted the usefulness of transcranial Doppler ultrasound as a simple, non-invasive method and suggest its potential as a screening tool for subtle CBF changes that may relate to HF-induced brain injury [23]. Brain structural abnormalities, including cerebral atrophy due to chronic regional hypoperfusion (in areas with poor collateral blood flow, such as the medial temporal lobe), white matter hyperintensities, gray matter reduction, and silent cerebral infarctions, are often reported in patients with HF who exhibit cognitive impairment. These alterations are frequently accompanied by chronic inflammation and activation of neurohormonal pathways, such as the renin–angiotensin–aldosterone system and the adrenergic system. Indeed, increased adrenergic signaling and inflammatory activity promote RyR2-mediated Ca^2+^ leakage in neurons, which negatively affects memory and cognitive performance. The resulting disruption of intracellular Ca^2+^ balance elevates mitochondrial Ca^2+^ levels, leading to oxidative stress and changes in the expression of genes implicated in cognition. Consistent with this mechanism, HF mice treated with the RyR2 stabilizer drug (S107) prevented cognitive deficits induced by HF [24]. In addition, comorbidities, including atrial fibrillation and cardiorenal syndrome, together with factors such as hyponatremia and hyperhomocysteinemia, often accompany HF and may compromise cerebral perfusion or trigger metabolic, neurohormonal inflammatory, and inflammatory alterations that impact cerebral function [25].

Given the prevalence of this condition, cognitive function should be routinely assessed in patients with HF. Accordingly, a recent meta-analysis, evaluating the prognostic impact of cognitive impairment in HF patients followed for more than 3 months, showed that cognitive frailty was associated with an 88% higher risk of all-cause mortality and a 53% higher risk of the composite endpoint of all-cause mortality and readmission compared with preserved cognitive function [26]. Early identification of cognitive deficits may enhance risk stratification and guide closer monitoring of patients at risk of suboptimal self-care and poor recognition of HF worsening symptoms [27,28]. To evaluate cognitive frailty in HF patients, several scores have been proposed, including the Mini-Mental State Examination (MMSE) and the MoCA. The Mini-Mental State Examination is the most frequently applied screening tool; however, it does not appear to be sufficiently sensitive to detect subtle cognitive deficits or to adequately assess executive function. In this context, the MoCA appears to be a more effective screening tool for cognitive impairment in patients with HF [29]. In an international multicenter study exploring sociodemographic and clinical factors associated with cognitive impairment in HF patients, older age, educational level, and exercise capacity emerged as predictors of overall cognitive impairment. Exercise capacity was positively associated with global cognition and most cognitive domains, highlighting its potential as a modifiable factor in HF [30]. In this regard, a pilot study reported that a combined aerobic and cognitive training program was associated with improved memory in patients with HF, reinforcing the evidence that greater functional capacity may support better neurocognitive functioning, potentially through increased CBF.

Evidence suggests a positive association between the Six Minute Walking Test (6MWT) performance and global cognitive function (as measured by the MMSE). Therefore, routine assessment of functional capacity using simple performance tests, such as the 6MWT, may identify individuals at risk of cognitive frailty at an early stage [31].

Non-pharmacological strategies that involved cognitive intervention, cognitive training combined with exercise, exercise training, and self-care management had a positive effect on cognition, physical performance, and depression levels in frail HF patients. Additional studies using formal outcome measures and longer follow-up periods are necessary to provide stronger indications.

## 5. Cognitive Impairment After Cardiac Surgery

Cardiac surgery requiring cardiopulmonary bypass (CPB) frequently results in neurocognitive complications that represent a significant clinical challenge in perioperative care. Postoperative delirium (POD) and postoperative cognitive dysfunction (POCD) are prevalent neurological syndromes after cardiac surgery, meaningfully changing patient recovery and long-term outcomes, as well as increased risk of mortality, functional decline and persistent cognitive dysfunction [32]. These complications occur in a substantial proportion of cardiac surgery patients, with delirium affecting 16–73% of patients depending on the patient population and assessment methodology, and representing one of the most frequent acute neurological complications after cardiac surgery with adverse impacts on surgical outcomes [33,34]. The development of POD within 24–72 h after surgery is characterized by acute disturbances in consciousness, attention, and cognition, and has a significant impact on patient prognosis, including increased length of hospital stay, readmission rates, and hospital costs [35]. Emerging evidence also supports the role of ‘prehabilitation’—multimodal interventions, including exercise and nutritional optimization—to increase physiological reserve and potentially mitigate the neuroinflammatory response triggered by CPB [36]. Preoperative cognitive impairment itself serves as a critical risk factor—elderly patients with preoperative cognitive dysfunction are at substantially elevated risk for postoperative problems after cardiac surgery, with cognitive dysfunction associated with an 8-fold increased risk of delirium, a 5% increase in absolute risk of major postoperative bleeding, and an increase in hospital and intensive care unit length of stay [37].

The pathophysiological mechanisms underlying cognitive dysfunction after cardiac surgery are multifactorial and complex, involving CPB-induced inflammation and cerebral injury. CPB initiates a cascade of pathophysiological changes that trigger systemic inflammation and potential neurological injury through several mechanisms, including ischemia-reperfusion injury, cerebral hypoperfusion, embolic events, and neuroinflammation [38]. Postoperative systemic inflammation, as assessed by inflammatory markers, plays a crucial role in the development of POD and POCD, with patients who developed POD/POCD exhibiting higher levels of IL-6 and the neutrophil-to-lymphocyte ratio (NLR) at 48 h postoperatively [32]. The blood–brain barrier dysfunction and neuroinflammatory response during CPB create conditions conducive to both acute delirium and potential long-term cognitive decline [39]. Advanced age (>70 years), prolonged CPB time (>120 min), hemodynamic instability, elevated postoperative lactate levels, maximum temperature, and high comorbidity index were strongly associated with neurological complications, including cognitive impairment [40,41]. Additional recognized clinical variables significantly associated with POD/POCD include extended mechanical ventilation, older age, renal dysfunction, vasopressor support duration, blood transfusion, elevated postoperative creatine kinase and lactate dehydrogenase, an ejection fraction less than 45%, and atrial fibrillation [32]. Importantly, elevated serum TDP-43 (TAR DNA-binding protein 43) levels increased significantly on postoperative day one compared to baseline, with this increase more pronounced in people who experienced delirium, suggesting that TDP-43 may be used as a prognostic biomarker for acute neurological problems and blood–brain barrier integrity after heart surgery [42].

Risk stratification and prevention strategies are essential for optimizing cognitive outcomes in cardiac surgery patients. Preoperative cognitive assessment using validated tools such as the MoCA is crucial, as cognitive testing has 87% sensitivity and 72% specificity in identifying cognitive impairment and has been associated with a 3-day longer hospital length of stay in cardiac surgery patients with identified impairment [43,44]. The coexistence of preoperative frailty and mild cognitive dysfunction poses a particular risk, with patients having both conditions demonstrating significantly higher odds ratios for developing delirium compared to those without these conditions [45]. Prevention and management strategies include both pharmacologic and non-pharmacologic approaches; dexmedetomidine demonstrated significant protective effects with reduced odds of delirium and higher MMSE scores compared to control groups [46,47]. Ciprofol, a novel sedative agent, significantly reduced the incidence of postoperative delirium compared with propofol, although no significant effect on postoperative cognitive dysfunction at 1 or 3 months was observed [48]. Gastrodin infusion significantly improved postoperative delirium incidence and enhanced discharge outcomes in patients undergoing CABG [49]. Furthermore, preoperative computerized cognitive training shows promise as a non-pharmacologic strategy, with the potential to reduce delirium incidence by stimulating cognitive domains in older cardiac surgery patients [35]. Long-term cognitive outcomes remain a significant concern, as postoperative delirium was associated with subjective cognitive decline and measured by validated instruments, up to 180 days after surgery, and preoperative cognitive impairment, combined with the duration of postoperative delirium, independently predicted decline in activities of daily living at six months follow-up [50,51]. Understanding these risk factors and implementing multidisciplinary approaches to prevention and management are essential for improving cognitive outcomes and overall recovery in this vulnerable population [52]. Ultimately, the persistence of these cognitive deficits into the recovery phase necessitates specialized screening within CR programs to tailor exercise prescriptions for those with lingering cognitive frailty.

## 6. Cardiac Rehabilitation and Cognitive Frailty

As reported, cognitive impairment is a common disorder following CVDs [53,54]; also, it seems cognitive impairments can influence patients’ treatment adherence, self-care, QOL, and secondary prevention programs [55].

### 6.1. Effects of Exercise and CR on Cognition

Firstly, some articles have shown the value of multidomain CR, including cognitive function training, in maintaining and improving cognition [56]. In addition, most studies showed strong cognitive improvement in attention/executive function and memory areas, particularly in patients without any type of heart surgery, while similar results were not reported in the language area. Nevertheless, this evidence should not be generalized due to the small number of data [57]. Moreover, data in the literature show possible improvements in cognitive functions both with multi-domain CR programs and with programs based only on physical exercise [56,58].

Indeed, in a randomized trial, Kwak et al. showed that regular exercise (30–60 min/day, 2–3 times per week for 12 months) enhanced cognition (MMSE score from 14.5 ± 5.3 to 17.5 ± 6.9 points) in participants with significant cognitive dysfunction [59]. Accordingly, Ngandu et al. reported the usefulness of a 2-year comprehensive program that counted regular exercise and cognitive training toward the enhancement of cognitive function assessed using a neuropsychological test series in older patients with dementia [58]. However, Fujiyoshi et al. showed that a CR intervention without cognitive training is useful in improving MMSE and Frontal Assessment Battery (FAB) scores [56]. This is probably possible due to the comprehensive and pleiotropic effects of CR [56].

Moreover, different exercise types and intensities can affect cognitive function differently. In the literature, a strong impact of structured aerobic exercise programs on the improvement in the executive function area in elderly patients in inpatient and outpatient settings was reported [60,61]. While the best type of exercise to improve cognition with CR is not clear, aerobic and resistance exercise of at least moderate intensity is supported by existing studies [62]. Indeed, engaging in 30–119 min of moderate-to-low physical activity per day seems to improve cognition, while higher intensity activities (from vigorous to maximal exercise) may have a negative effect, as they can disrupt metabolism in the frontal cortex of the brain in adults with heart disease [63]. Specifically, studies supporting exercise for cognitive benefits were carried out in supervised settings, both inpatient and outpatient. Strategies to implement CR for cognitive benefits effectively as part of home-based models is an area to explore [64].

Specifically, the frequency of sessions may also influence cognitive enhancement in the course of the CR program [57]. Indeed, in the article by Tanne et al. [65], with a frequency of two times per week, most outcome parameters did not reach a significant improvement in the course of CR. Conversely, Cavalcante et al. showed that a three-times per day physiotherapy session can improve cognition better than a one-time per day session in phase I CR [66]. Consequently, the data of this study show that CR can enhance cognition in CVD patients with at least three times per week frequency, but such improvement has not been reported in the language area [57].

**Figure 1:**
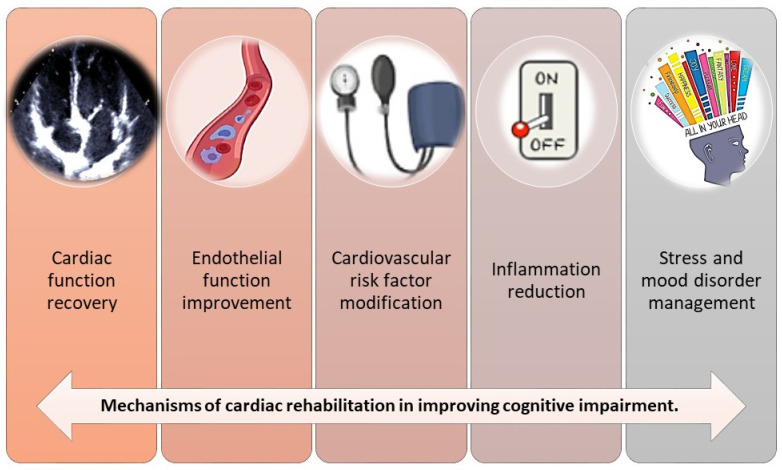
Mechanisms of cardiac rehabilitation in improving cognitive impairment.

Although no univocal mechanism has been identified for the effects of CR on cognitive performance, several possible mechanisms have been proposed (Figure 1) [57]. Moreover, evidence recorded in murine models of cardiac stress showed that exercise exerts beneficial CV effects by regulating some epigenetic mechanisms [67]. Precisely, some non-coding RNAs (microRNAs and long non-coding RNAs) were significantly modified by exercise [67].

Generally, it seems that exercise can increase cardiac output and promote cardiac function recovery, ultimately growing CBF, which is one of the aspects related to cognitive dysfunction [68,69]. Second, cognitive frailty is associated with some cardiovascular factors such as blood pressure and cardiac inflammatory markers; therefore, CR can expand the cognitive area through modification of these parameters [70,71].

Also, it is proposed that improved cardiovascular fitness and exercise capacity achieved by CR influence cognition [72]. Precisely, the improvement in endothelial function may lead to the enhancement of cognition [56]. Accordingly, Fujiyoshi et al. showed that the modification in the RH-PAT index was greater in the CR group than in the control group [56]. Indeed, the improvement in endothelial function may lead to the maintenance of cognitive function through recovery of the cerebral microcirculation via enhanced nitric oxide synthesis, prostacyclin, tissue plasminogen activator, and shear stress [73]. Moreover, exercise acts to stimulate the secretion of brain-derived neurotrophic factor, which is associated with neurogenesis, synaptogenesis, and dendritogenesis [74]. Also, exercise promotes mitochondrial function, which plays a neuroprotective role through both brain plasticity and angiogenesis [75].

Obviously, the modification of CV risk factors is also one of the mechanisms underlying the improvement in cognitive functions; precisely, improved blood pressure levels and metabolic profiles (management of metabolic syndrome, obesity, and diabetes), often addressed through nutritional counseling in CR, directly benefit brain health [76].

Also, inflammatory biomarkers (e.g., c-reactive protein) have been linked to cognitive dysfunction and CVD [70], while improvements in inflammatory pathways may lead to improvements in cognitive domains [69]. Precisely, myokines, which are released from skeletal muscle during exercise, play a crucial role in these anti-inflammatory effects and promote inter-tissue communication and cardiovascular benefits [77,78].

Finally, it is reported that the psychological effects of CR (increased activity, optimism, and improved exercise habits) may lead to the reported benefits in the cognition area by elevating dopamine levels [5].

### 6.2. CR in Patients with Cognitive Frailty: How to Implement It

Cardiac guidelines emphasize that cognitive dysfunction is a comorbidity of cardiac disease [79]. However, actually, there are no specific evidence-based cognitive screening strategies for cardiac patients. Nevertheless, early detection of cognitive impairment in this population may guide clinical strategies.

CR programs include a multidomain cardiovascular evaluation of the patient, cardiovascular risk factor modification, supervised training exercises, advice on physical activity and nutrition, and psychosocial support [80]. Regrettably, CR is underutilized, and participation gaps and barriers are often seen in programs [81]. These gaps and barriers are especially evident in frailty patients (Table 1).

Enrollment in outpatient CR was linked to lower rates of composite events and enhanced physical function in participants with CVDs, regardless of cognitive frailty. Consequently, promoting active participation in CR, even among adults with cognitive frailty, is indispensable as it is linked to positive clinical outcomes. Moreover, even if these participants are unable to attend all expected CR sessions, studies have shown an incremental benefit of individual sessions on hospitalizations.

By evaluating cognitive frailty for risk stratification and operationalizing CR programs, healthcare providers can help these participants improve prognosis and reduce the risk of other complications by treating their current cognitive frailty [89]. These approaches could improve CR completion rates among patients with cognitive frailty. Finally, we must also define whether electronic and remote delivery of information and care are reliable for this specific population [90]. Indeed, digital health strategies have been previously used in CR and for frail patients [91,92]; nevertheless, further research in this area may assist traditional CR programs in adapting to participants’ needs and accessibility.

Also, there has been attention in pharmacotherapy that may slow cognitive dysfunction and prevent or improve cognitive frailty. There is weak evidence that the omega-3 polyunsaturated fatty acids docosahexaenoic acid and eicosapentaenoic acid may be neuroprotective and decrease the incidence of cognitive dysfunction [89]. A pilot study was conducted to assess the impact of Efalex Active 50+ supplement, which contains 1 g docosahexaenoic acid and 160 mg eicosapentaenoic acid in addition to phosphatidylserine, Ginkgo biloba, alpha-tocopherol, vitamin B12, and folic acid, among elderly patients [93]. Although these are weak associations, the participants in the intervention group had a mild enhancement in some domains of cognition and mobility. However, the role of this supplementation is yet to be confirmed in a larger trial.

Finally, the association between certain dietary patterns and cognitive impairment was explored, but significant confounding and selection bias existed. For example, the Mediterranean diet has been linked to a decreased possibility of cognitive decline [85], possibly because of a higher antioxidant content (beta-carotene equivalents, vitamin C, and vitamin E), which, through their anti-inflammatory properties, may also help prevent cognitive frailty. However, it is hard to establish a causal association between nutritional strategies and improvements in cognitive frailty because of bias, but certain nutritional interventions (e.g., the Mediterranean diet, protein supplementation combined with physical intervention) may affect cognitive impairment in patients with CVD.

## 7. Limitations, Gaps in Evidence, and Future Perspectives

As reported, cognitive impairment is frequently observed in a variety of cardiovascular conditions, including HF, hypertension, CABG, and post-sudden cardiac arrest survival, and is generally recognized as a risk marker for adverse outcomes in these patients. On the other hand, CR, an increasingly recognized therapeutic intervention in CVDs, has been reported to improve clinical outcomes in patients with cognitive impairment. Growing evidence suggests that its positive effects on cognitive function are mediated through multiple mechanisms, including increased cerebral blood flow, improved vascular function, and neuroplastic changes in brain regions involved in cognition. However, many gaps in the evidence remain to be addressed (Table 2).

This review is subject to numerous limitations due to the available literature: heterogeneity in CR duration, structure, outcome measures, and study quality ruled out formal meta-analysis; moreover, many studies were observational, raising the potential for bias and confounding, and the study sample sizes are often small and follow-up is short; also, cognitive screening questionnaires, while validated, were not invariably used, making direct comparisons difficult.

Precisely, current evidence remains largely indirect and based on observational data. This highlights the need for future studies to integrate MRI brain imaging, vascular and inflammatory biomarkers, and exercise physiology measures. For example, by assessing subcortical brain volume using MRI alongside cognitive function through neuropsychological testing, researchers have found that exercise in older adults with HF is associated with improvements in both brain structure and cognitive performance [96].

To better explore the association between cognition and CR, high-quality randomized controlled trials with long-term follow-up assessing cognitive trajectories are needed. Patients with cognitive deficits are frequently excluded from clinical studies, despite the high prevalence of cognitive dysfunction in cardiovascular populations. Current JCS/JACR guidelines [95] recommend that rehabilitation programs incorporate not only exercise training but also cognitive interventions, psychological counseling, stress management, and patient education. However, cognitive assessment is still rarely integrated into standard CR protocols, highlighting a critical gap between guideline recommendations and routine clinical practice. It is important to routinely assess the mental and psychological status of patients approaching CR using questionnaire-based screening tests. Regarding mild cognitive impairment and dementia, it is difficult to rule out these conditions based solely on traditional screening tests. Therefore, a systematic cognitive screening program covering all cognitive domains, and potentially including imaging tests, is necessary. For example, a subgroup analysis on all-cause mortality in patients with HF and cognitive impairment showed that cognitive deficits defined by the MMSE or MoCA exhibited different prognostic value, while impairment detected by the Mini-Cog appeared to provide additive prognostic information compared with the MMSE. On the other hand, we recently used the Italian version of the Qmci screen in a CR population [9]; precisely, this test is useful for MCI, because of its higher sensitivity compared with the standardized MMSE and ABCS 135 in differentiating MCI from normal cognition [97]. However, due to the limited number of studies, it is not possible to determine which tool is superior. Additional studies are required to directly compare the prognostic role of cognitive impairment as defined by different screening questionnaires [26].

Another important issue is that most studies evaluate cognitive outcomes only during or shortly after rehabilitation, while the long-term impact of CR on cognitive decline remains largely unexplored. Consequently, longitudinal cohort studies with extended follow-up are needed to better characterize the long-term effects of CR on cognitive function.

Finally, effectively addressing cognitive impairment requires a multidisciplinary, team-based approach. This should involve coordinated delivery of multidomain interventions that combine exercise training, cognitive rehabilitation, nutritional support, and psychosocial care [25]. Moreover, to date, some gaps persist in the implementation of CR integration with current guideline-directed treatments, including device-based and pharmacological advances [98].

Future research should focus on evaluating the effectiveness of integrated cognitive–cardiovascular rehabilitation programs to optimize both cardiac and cognitive outcomes.

## 8. Conclusions

In conclusion, maintaining cognitive function is a crucial factor in secondary prevention and can be a therapeutic and interventional target in patients with CVD. The simple incorporation of cognitive impairment screening into phase I of CR provides prognostic information for participants. Recognizing patients with cognitive problems delivers a chance to intervene, which may slow the progression of their cognitive frailty. Specifically, the addition of appropriate nutritional strategies and cognitive activities (games, music, reading, etc.) is necessary to build a more effective, exercise-based, comprehensive CR that may improve cognition in participants with CVD.

Poor cardiorespiratory fitness is also linked to cognitive frailty, with CR being associated with enhancements in cognitive function; consequently, referral to phase II of CR will allow them to derive benefits that may improve their cognitive function, in addition to providing the typical benefits of CR.

Future CR programs should explore unconventional delivery models, including home-based programs, to expand access and improve compliance of patients with cognitive frailty. High-quality trials are needed to improve CR strategies and guarantee long-term benefits across this population.

## Figures and Tables

**Table 1:** CR barriers and possible solutions in frailty patients.

Barrier	Solution
Underestimation of the prevalence of cognitive frailty in CR patients [55]	Because CV procedures and events can affect cognition, incorporation of cognitive impairment screening into phase I of CR is fundamental [82].
Inability to attend all prescribed CR sessions [83]	There is, however, an incremental benefit of individual sessions on readmission rates because CR has a dose–response relationship [84].
Logistical and organizational difficulties for the patient and the caregiver: e.g., socioeconomic factors, travel and transportation [83].	Alternative care delivery models need to be considered. Accordingly, it is essential to offer flexible schedules, home-based CR options and digital health interventions, also to accommodate caregiving roles [83].
Presence of malnutrition or cachexia [9]	Offer nutritional support within the CR program, with diets rich in antioxidants (e.g., Mediterranean diet) [85]. Moreover, nutritional content applications enable patients to log their dietary intake and have direct feedback on modifications that can be made [83].
Lack of awareness about the need for CR [86].	Educate patients and caregivers about the importance of CR and its long-term benefits. Specifically, physician recommendation for CR participation is one of the strongest. predictors for patient enrolment, especially in women and the elderly [87,88].

**Table 2:** Gap in evidence and future perspectives.

Gap in Evidence	Future Perspectives
Current evidence remains largely indirect and based on observational data, and patients with cognitive deficits are frequently excluded from clinical studies [94].	Need to integrate MRI brain imaging, vascular and inflammatory biomarkers, and exercise physiology measures. Need for randomized controlled trials with long-term follow-up.
Despite current CR guidelines [95], cognitive assessment is still rarely integrated into standard CR protocols [55].	It is necessary to have a systematic cognitive screening program covering all cognitive domains, and potentially including imaging tests.
Due to the limited number of studies, it is not possible to determine which cognitive screening tool is superior [26].	Additional studies are required to directly compare different cognitive screening tools in this specific population.

## Data Availability

No new data were created or analyzed in this study. Data sharing is not applicable to this article.

## Abbreviations

The following abbreviations are used in this manuscript:

6MWT	Six Minute Walking Test
BDNF	Brain-Derived Neurotrophic Factor
CABG	Coronary artery bypass grafting
CAD	Coronary artery disease
CBF	Cerebral blood flow
CFR	Coronary flow reserve
CMRO_2_	Cerebral metabolic rate of oxygen
CPB	Cardiopulmonary bypass
CR	Cardiac rehabilitation
CSVD	Cerebral small vessel disease
CVDs	Cardiovascular diseases
FAB	Frontal Assessment Battery
HF	Heart failure
MCI	Mild cognitive impairment
METs	Metabolic Equivalent of Task
MI	Myocardial infarction
MMSE	Mini-Mental State Examination
MoCA	Montreal Cognitive Assessment
MRI	Magnetic resonance imaging
NLR	Neutrophil-to-lymphocyte ratio
OEF	Oxygen extraction fraction
PCI	Percutaneous coronary intervention
POCD	Postoperative cognitive dysfunction
POD	Postoperative delirium
Qmci	Quick Mild Cognitive Impairment
QOL	Quality of life

## References

[1] Goh F.Q., Tan B.Y.Q., Yeo L.L.L., Sia C.H. (2025). The Heart-Brain Axis: Key Concepts in Neurocardiology. Cardiol. Discov..

[2] Kerola T., Kettunen R., Nieminen T. (2011). The complex interplay of cardiovascular system and cognition: How to predict dementia in the elderly?. Int. J. Cardiol..

[3] Grzybowska-Ganszczyk D., Nowak Z., Opara J.A., Nowak-Lis A. (2025). Hypoxia and Cognitive Functions in Patients Suffering from Cardiac Diseases: A Narrative Review. J. Clin. Med..

[4] Ni F., Mi W., Wei Y., Li Z., Fan Y., Xi Z. (2025). Evidence Summary of Management Strategies for Cognitive Impairment in Patients with Heart Failure. J. Multidiscip. Healthc..

[5] Zergaw M., Elgendy M., Billey A., Saleem A., Zeeshan B., Dissanayake G., Nassar S. (2024). The Long-Term Impact of Cardiac Rehabilitation on Cognitive Function in Older Patients With Myocardial Infarction: A Systematic Review. Cureus.

[6] Intzandt B., Black S.E., Lanctot K.L., Herrmann N., Oh P., Middleton L.E. (2015). Is Cardiac Rehabilitation Exercise Feasible for People with Mild Cognitive Impairment?. Can. Geriatr. J..

[7] Gunstad J., Macgregor K.L., Paul R.H., Poppas A., Jefferson A.L., Todaro J.F., Cohen R.A. (2005). Cardiac rehabilitation improves cognitive performance in older adults with cardiovascular disease. J. Cardiopulm. Rehabil..

[8] Gaalema D.E., Mahoney K., Ballon J.S. (2021). Cognition and Exercise: General Overview and Implications for Cardiac Rehabilitation. J. Cardiopulm. Rehabil. Prev..

[9] Virtuoso N., Palmieri F., Loria F., Squillante A., Izzo C., Fortunato M., Fiorentino F., Sparano E., Luca A., Fucile I. (2026). Analysis of a Real-World Population Participating in a Cardiac Rehabilitation Program: Cognitive Impairment, Functional Capacity, and Therapy Titration. J. Clin. Med..

[10] Besnier F., Dupuy E.G., Gagnon C., Vincent T., Vrinceanu T., Blanchette C.A., Iglesies-Grau J., Saillant K., Chabot-Blanchet M., Belleville S. (2025). Effects of home-based exercise with or without cognitive training on cognition and mobility in cardiac patients: A randomized clinical trial. Geroscience.

[11] Ishihara K., Izawa K.P., Kitamura M., Kanejima Y., Ogawa M., Yoshihara R., Morisawa T., Shimizu I. (2024). Effects of cardiac rehabilitation on cognitive function in patients with acute coronary syndrome: A systematic review. Heliyon.

[12] Wolters F.J., Segufa R.A., Darweesh S.K.L., Bos D., Ikram M.A., Sabayan B., Hofman A., Sedaghat S. (2018). Coronary heart disease, heart failure, and the risk of dementia: A systematic review and meta-analysis. Alzheimer’s Dement..

[13] Jiang X., O’Bryant S.E., Johnson L.A., Rissman R.A., Yaffe K. (2023). The Health and Aging Brain Study (HABS-HD) Study Team. Association of cardiovascular risk factors and blood biomarkers with cognition: The HABS-HD study. Alzheimer’s Dement..

[14] Cox S.R., Lyall D.M., Ritchie S.J., Bastin M.E., Harris M.A., Buchanan C.R., Fawns-Ritchie C., Barbu M.C., de Nooij L., Reus L.M. (2019). Associations between vascular risk factors and brain MRI indices in UK Biobank. Eur. Heart J..

[15] Mejia-Renteria H., Travieso A., Matias-Guiu J.A., Yus M., Espejo-Paeres C., Finocchiaro F., Fernandez S., Gomez-Escalonilla C.I., Reneses-Prieto B., Gomez-Garre M.D. (2023). Coronary microvascular dysfunction is associated with impaired cognitive function: The Cerebral-Coronary Connection study (C3 study). Eur. Heart J..

[16] Rosengart T.K., Sweet J., Finnin E.B., Wolfe P., Cashy J., Hahn E., Marymont J., Sanborn T. (2005). Neurocognitive functioning in patients undergoing coronary artery bypass graft surgery or percutaneous coronary intervention: Evidence of impairment before intervention compared with normal controls. Ann. Thorac. Surg..

[17] Li B., Lu X., Moeini M., Sakadzic S., Thorin E., Lesage F. (2019). Atherosclerosis is associated with a decrease in cerebral microvascular blood flow and tissue oxygenation. PLoS ONE.

[18] Xie W., Zheng F., Yan L., Zhong B. (2019). Cognitive Decline Before and After Incident Coronary Events. J. Am. Coll. Cardiol..

[19] Liang J., Li C., Gao D., Ma Q., Wang Y., Pan Y., Zhang W., Xie W., Zheng F. (2023). Association Between Onset Age of Coronary Heart Disease and Incident Dementia: A Prospective Cohort Study. J. Am. Heart Assoc..

[20] Sanami S., Tremblay S.A., Rezaei A., Potvin-Jutras Z., Sabra D., Intzandt B., Gagnon C., Mainville-Berthiaume A., Wright L., Gayda M. (2025). The Impact of Coronary Artery Disease on Brain Vascular and Metabolic Health: Links to Cognitive Function. Aging Dis..

[21] Thorp E.B., Flanagan M.E., Popko B., DeBerge M. (2022). Resolving inflammatory links between myocardial infarction and vascular dementia. Semin. Immunol..

[22] Zhao Q., Liu X., Wan X., Yu X., Cao X., Yang F., Cai Y. (2023). Non-pharmacological interventions for cognitive impairment in older adults with heart failure: A systematic review. Geriatr. Nurs..

[23] Ovsenik A., Podbregar M., Fabjan A. (2021). Cerebral blood flow impairment and cognitive decline in heart failure. Brain Behav..

[24] Dridi H., Liu Y., Reiken S., Liu X., Argyrousi E.K., Yuan Q., Miotto M.C., Sittenfeld L., Meddar A., Soni R.K. (2023). Heart failure-induced cognitive dysfunction is mediated by intracellular Ca^2+^ leak through ryanodine receptor type 2. Nat. Neurosci..

[25] Liori S., Arfaras-Melainis A., Bistola V., Polyzogopoulou E., Parissis J. (2022). Cognitive impairment in heart failure: Clinical implications, tools of assessment, and therapeutic considerations. Heart Fail. Rev..

[26] Zhang H., Jie Y., Sun Y., Wang X., Gong D., Fan Y. (2022). Association of Cognitive Impairment With Mortality and Readmission in Patients With Heart Failure: A Meta-analysis. Curr. Probl. Cardiol..

[27] Visco V., Esposito C., Vitillo P., Vecchione C., Ciccarelli M. (2020). It is easy to see, but it is better to foresee: A case report on the favourable alliance between CardioMEMS and levosimendan. Eur. Heart J. Case Rep..

[28] Visco V., Esposito C., Manzo M., Fiorentino A., Galasso G., Vecchione C., Ciccarelli M. (2022). A Multistep Approach to Deal With Advanced Heart Failure: A Case Report on the Positive Effect of Cardiac Contractility Modulation Therapy on Pulmonary Pressure Measured by CardioMEMS. Front. Cardiovasc. Med..

[29] Davis K.K., Allen J.K. (2013). Identifying cognitive impairment in heart failure: A review of screening measures. Heart Lung.

[30] Vellone E., Chiala O., Boyne J., Klompstra L., Evangelista L.S., Back M., Ben Gal T., Martensson J., Stromberg A., Jaarsma T. (2020). Cognitive impairment in patients with heart failure: An international study. ESC Heart Fail..

[31] Gary R.A., Paul S., Corwin E., Butts B., Miller A.H., Hepburn K., Williams B., Waldrop-Valverde D. (2019). Exercise and Cognitive Training as a Strategy to Improve Neurocognitive Outcomes in Heart Failure: A Pilot Study. Am. J. Geriatr. Psychiatry.

[32] Staicu R.E., Vernic C., Ciurescu S., Lascu A., Aburel O.M., Deutsch P., Rosca E.C. (2025). Postoperative Delirium and Cognitive Dysfunction After Cardiac Surgery: The Role of Inflammation and Clinical Risk Factors. Diagnostics.

[33] Teller J., Gabriel M.M., Schimmelpfennig S.D., Laser H., Lichtinghagen R., Schafer A., Fegbeutel C., Weissenborn K., Jung C., Hinken L. (2022). Stroke, Seizures, Hallucinations and Postoperative Delirium as Neurological Complications after Cardiac Surgery and Percutaneous Valve Replacement. J. Cardiovasc. Dev. Dis..

[34] Lechowicz K., Szylinska A., Cecerska-Heryc E., Ostrycharz-Jasek E., Zagrodnik E., Pacholewicz J., Dolegowska B., Kotfis K. (2025). Association of Plasma BDNF Concentration and Val66Met Polymorphism with Postoperative Delirium After Cardiac Surgery Under General Anesthesia with Cardiopulmonary Bypass. J. Clin. Med..

[35] Qiu X., Wang L., Wen X., Meng Q., Qi J., Li C., Yin H., Ling F., Yuhan Q., Zhang W. (2024). Effect of different durations of preoperative computerised cognitive training on postoperative delirium in older patients undergoing cardiac surgery: A study protocol for a prospective, randomised controlled trial. BMJ Open.

[36] Gillis C., Ljungqvist O., Carli F. (2022). Prehabilitation, enhanced recovery after surgery, or both? A narrative review. Br. J. Anaesth..

[37] Au E., Thangathurai G., Saripella A., Yan E., Englesakis M., Nagappa M., Chung F. (2023). Postoperative Outcomes in Elderly Patients Undergoing Cardiac Surgery With Preoperative Cognitive Impairment: A Systematic Review and Meta-Analysis. Anesth. Analg..

[38] Zhao N., Qin R., Liu B., Zhang D. (2025). Sevoflurane versus propofol on immediate postoperative cognitive dysfunction in patients undergoing cardiac surgery under cardiopulmonary bypass: A comparative analysis. J. Cardiothorac. Surg..

[39] Di L., Huang P., He Y., Li J., Liu Y., Chi L., Sun N., Huang L. (2025). Association between preoperative blood-brain barrier permeability and postoperative delirium in older patients undergoing cardiac surgery: A pilot study. Aging Clin. Exp. Res..

[40] Fang J., Liang H., Chen M., Zhao Y., Wei L. (2024). Association of preoperative cognitive frailty with postoperative complications in older patients under general anesthesia: A prospective cohort study. BMC Geriatr..

[41] Zhao A., Peng Y., Lin L., Chen L., Lin Y. (2025). The influencing factors of cognitive dysfunction in patients after cardiac surgery and the construction of a nomogram prediction model. Eur. J. Med. Res..

[42] Simon C., Graves O.K., Akeju O., McKay T.B. (2025). Elevated TDP-43 serum levels associated with postoperative delirium following major cardiac surgery. Brain Behav. Immun. Health.

[43] Danquah M.O., Yan E., Lee J.W., Philip K., Saripella A., Alhamdah Y., He D., Englesakis M., Chung F. (2024). The utility of the Montreal cognitive assessment (MoCA) in detecting cognitive impairment in surgical populations—A systematic review and meta-analysis. J. Clin. Anesth..

[44] Corbett P.F.M., Sadoun A., Yoshimatsu Y., Martin F.E., Braude P., Avlonitis V.S., Partridge J.S.L., Dhesi J.K. (2026). Five-Year Functional and Cognitive Trajectories Following Cardiac Surgery in Older Adults: A Prospective Study. J. Am. Med. Dir. Assoc..

[45] Itagaki A., Sakurada K., Matsuhama M., Yajima J., Yamashita T., Kohzuki M. (2020). Impact of frailty and mild cognitive impairment on delirium after cardiac surgery in older patients. J. Cardiol..

[46] Neerland B.E., Busund R., Haaverstad R., Helbostad J.L., Landsverk S.A., Martinaityte I., Norum H.M., Raeder J., Selbaek G., Simpson M.R. (2022). Alpha-2-adrenergic receptor agonists for the prevention of delirium and cognitive decline after open heart surgery (ALPHA2PREVENT): Protocol for a multicentre randomised controlled trial. BMJ Open.

[47] Qureshi O., Arthur M.E. (2023). Recent advances in predicting, preventing, and managing postoperative delirium. Fac. Rev..

[48] Guo P., Li X., Ren C., Duan Z., Kong Y., Bi M., Liu F., Wang Y., Chen L., Zhang Y. (2026). The effect of ciprofol on the incidence of postoperative delirium in adult surgical patients: A meta-analysis and meta-regression. Front. Neurol..

[49] Bai Y.X., Wu H.L., Xie W.L., Li X., Han J.J., Liu J., Chen S.Q., Yin P., Dong N.G., Wu Q.P. (2025). Efficacy and safety of gastrodin in preventing postoperative delirium following cardiac surgery: A randomized placebo controlled clinical trial. Crit. Care.

[50] Guenther U., Hoffmann F., Dewald O., Malek R., Brimmers K., Theuerkauf N., Putensen C., Popp J. (2020). Preoperative Cognitive Impairment and Postoperative Delirium Predict Decline in Activities of Daily Living after Cardiac Surgery—A Prospective, Observational Cohort Study. Geriatrics.

[51] Namirembe G.E., Baker S., Albanese M., Mueller A., Qu J.Z., Mekonnen J., Wiredu K., Westover M.B., Houle T.T., Akeju O. (2023). Association Between Postoperative Delirium and Long-Term Subjective Cognitive Decline in Older Patients Undergoing Cardiac Surgery: A Secondary Analysis of the Minimizing Intensive Care Unit Neurological Dysfunction with Dexmedetomidine-Induced Sleep Trial. J. Cardiothorac. Vasc. Anesth..

[52] Cohen C.L., Atkins K.J., Evered L.A., Silbert B.S., Scott D.A. (2023). Examining Subjective Psychological Experiences of Postoperative Delirium in Older Cardiac Surgery Patients. Anesth. Analg..

[53] Burkauskas J., Lang P., Bunevicius A., Neverauskas J., Buciute-Jankauskiene M., Mickuviene N. (2018). Cognitive function in patients with coronary artery disease: A literature review. J. Int. Med. Res..

[54] Gorelick P.B., Scuteri A., Black S.E., Decarli C., Greenberg S.M., Iadecola C., Launer L.J., Laurent S., Lopez O.L., Nyenhuis D. (2011). Vascular contributions to cognitive impairment and dementia: A statement for healthcare professionals from the american heart association/american stroke association. Stroke.

[55] Salzwedel A., Heidler M.D., Haubold K., Schikora M., Reibis R., Wegscheider K., Jobges M., Voller H. (2017). Prevalence of mild cognitive impairment in employable patients after acute coronary event in cardiac rehabilitation. Vasc. Health Risk Manag..

[56] Fujiyoshi K., Minami Y., Yamaoka-Tojo M., Kutsuna T., Obara S., Aoyama A., Ako J. (2020). Effect of cardiac rehabilitation on cognitive function in elderly patients with cardiovascular diseases. PLoS ONE.

[57] Dabbaghipour N., Javaherian M., Moghadam B.A. (2021). Effects of cardiac rehabilitation on cognitive impairments in patients with cardiovascular diseases: A systematic review. Int. J. Neurosci..

[58] Ngandu T., Lehtisalo J., Solomon A., Levalahti E., Ahtiluoto S., Antikainen R., Backman L., Hanninen T., Jula A., Laatikainen T. (2015). A 2 year multidomain intervention of diet, exercise, cognitive training, and vascular risk monitoring versus control to prevent cognitive decline in at-risk elderly people (FINGER): A randomised controlled trial. Lancet.

[59] Kwak Y.S., Um S.Y., Son T.G., Kim D.J. (2008). Effect of regular exercise on senile dementia patients. Int. J. Sports Med..

[60] Saez de Asteasu M.L., Martinez-Velilla N., Zambom-Ferraresi F., Casas-Herrero A., Cadore E.L., Galbete A., Izquierdo M. (2019). Assessing the impact of physical exercise on cognitive function in older medical patients during acute hospitalization: Secondary analysis of a randomized trial. PLoS Med..

[61] Baker L.D., Frank L.L., Foster-Schubert K., Green P.S., Wilkinson C.W., McTiernan A., Plymate S.R., Fishel M.A., Watson G.S., Cholerton B.A. (2010). Effects of aerobic exercise on mild cognitive impairment: A controlled trial. Arch. Neurol..

[62] Northey J.M., Cherbuin N., Pumpa K.L., Smee D.J., Rattray B. (2018). Exercise interventions for cognitive function in adults older than 50: A systematic review with meta-analysis. Br. J. Sports Med..

[63] Dong X., Yi X., Jia N., Ding M., Zhou Y., Tian C. (2022). Associations of physical activity with cognitive function and daily physical function among Chinese individuals with heart disease: A cross-sectional study. Front. Public Health.

[64] Alfaraidhy M.A., Regan C., Forman D.E. (2022). Cardiac rehabilitation for older adults: Current evidence and future potential. Expert Rev. Cardiovasc. Ther..

[65] Tanne D., Freimark D., Poreh A., Merzeliak O., Bruck B., Schwammenthal Y., Schwammenthal E., Motro M., Adler Y. (2005). Cognitive functions in severe congestive heart failure before and after an exercise training program. Int. J. Cardiol..

[66] Cavalcante E.D., Magario R., Conforti C.A., Cipriano Junior G., Arena R., Carvalho A.C., Buffolo E., Luna Filho B. (2014). Impact of Intensive Physiotherapy on Cognitive Function after Coronary Artery Bypass Graft Surgery. Arq. Bras. Cardiol..

[67] Visco V., Forte M., Giallauria F., D’Ambrosio L., Piccoli M., Schiattarella G.G., Mancusi C., Salerno N., Cesaro A., Perrone M.A. (2025). Epigenetic mechanisms underlying the beneficial effects of cardiac rehabilitation. An overview from the working groups of “cellular and molecular biology of the heart” and “cardiac rehabilitation and cardiovascular prevention” of the Italian Society of Cardiology (SIC). Int. J. Cardiol..

[68] Cannon J.A., McMurray J.J., Quinn T.J. (2015). Hearts and minds’: Association, causation and implication of cognitive impairment in heart failure. Alzheimer’s Res. Ther..

[69] Stanek K.M., Gunstad J., Spitznagel M.B., Waechter D., Hughes J.W., Luyster F., Josephson R., Rosneck J. (2011). Improvements in cognitive function following cardiac rehabilitation for older adults with cardiovascular disease. Int. J. Neurosci..

[70] Beeri M.S., Ravona-Springer R., Silverman J.M., Haroutunian V. (2009). The effects of cardiovascular risk factors on cognitive compromise. Dialogues Clin. Neurosci..

[71] Taylor R.S., Brown A., Ebrahim S., Jolliffe J., Noorani H., Rees K., Skidmore B., Stone J.A., Thompson D.R., Oldridge N. (2004). Exercise-based rehabilitation for patients with coronary heart disease: Systematic review and meta-analysis of randomized controlled trials. Am. J. Med..

[72] Garcia S., Alosco M.L., Spitznagel M.B., Cohen R., Raz N., Sweet L., Josephson R., Hughes J., Rosneck J., Oberle M.L. (2013). Cardiovascular fitness associated with cognitive performance in heart failure patients enrolled in cardiac rehabilitation. BMC Cardiovasc. Disord..

[73] Verma S., Wang C.H., Li S.H., Dumont A.S., Fedak P.W., Badiwala M.V., Dhillon B., Weisel R.D., Li R.K., Mickle D.A. (2002). A self-fulfilling prophecy: C-reactive protein attenuates nitric oxide production and inhibits angiogenesis. Circulation.

[74] Coelho F.G., Vital T.M., Stein A.M., Arantes F.J., Rueda A.V., Camarini R., Teodorov E., Santos-Galduroz R.F. (2014). Acute aerobic exercise increases brain-derived neurotrophic factor levels in elderly with Alzheimer’s disease. J. Alzheimer’s Dis..

[75] Burtscher J., Millet G.P., Place N., Kayser B., Zanou N. (2021). The Muscle-Brain Axis and Neurodegenerative Diseases: The Key Role of Mitochondria in Exercise-Induced Neuroprotection. Int. J. Mol. Sci..

[76] Damluji A.A., Tomczak C.R., Hiser S., O’Neill D.E., Goyal P., Pack Q.R., Foulkes S.J., Brown T.M., Haykowsky M.J., Needham D.M. (2025). Benefits of Cardiac Rehabilitation: Mechanisms to Restore Function and Clinical Impact. Circ. Res..

[77] Cuccurullo S.J., Fleming T.K., Petrosyan H., Hanley D.F., Raghavan P. (2024). Mechanisms and benefits of cardiac rehabilitation in individuals with stroke: Emerging role of its impact on improving cardiovascular and neurovascular health. Front. Cardiovasc. Med..

[78] Huang C. (2023). Effect of new cardiac rehabilitation mode on cardiac function, mental state and quality of life of postoperative patients with acute myocardial infarction treated with atorvastatin calcium tablet. Eur. Rev. Med. Pharmacol. Sci..

[79] McDonagh T.A., Metra M., Adamo M., Gardner R.S., Baumbach A., Bohm M., Burri H., Butler J., Celutkiene J., Chioncel O. (2022). 2021 ESC Guidelines for the diagnosis and treatment of acute and chronic heart failure: Developed by the Task Force for the diagnosis and treatment of acute and chronic heart failure of the European Society of Cardiology (ESC). With the special contribution of the Heart Failure Association (HFA) of the ESC. Eur. J. Heart Fail.

[80] Piepoli M.F., Corra U., Benzer W., Bjarnason-Wehrens B., Dendale P., Gaita D., McGee H., Mendes M., Niebauer J., Zwisler A.D. (2010). Secondary prevention through cardiac rehabilitation: From knowledge to implementation. A position paper from the Cardiac Rehabilitation Section of the European Association of Cardiovascular Prevention and Rehabilitation. Eur. J. Cardiovasc. Prev. Rehabil..

[81] Thomas R.J. (2007). Cardiac rehabilitation/secondary prevention programs: A raft for the rapids: Why have we missed the boat?. Circulation.

[82] van Nieuwkerk A.C., Delewi R., Wolters F.J., Muller M., Daemen M., Biessels G.J., Heart-Brain Connection C. (2023). Cognitive Impairment in Patients With Cardiac Disease: Implications for Clinical Practice. Stroke.

[83] Chindhy S., Taub P.R., Lavie C.J., Shen J. (2020). Current challenges in cardiac rehabilitation: Strategies to overcome social factors and attendance barriers. Expert Rev. Cardiovasc. Ther..

[84] Martin B.J., Hauer T., Arena R., Austford L.D., Galbraith P.D., Lewin A.M., Knudtson M.L., Ghali W.A., Stone J.A., Aggarwal S.G. (2012). Cardiac rehabilitation attendance and outcomes in coronary artery disease patients. Circulation.

[85] Feart C., Samieri C., Rondeau V., Amieva H., Portet F., Dartigues J.F., Scarmeas N., Barberger-Gateau P. (2009). Adherence to a Mediterranean diet, cognitive decline, and risk of dementia. JAMA.

[86] Ades P.A., Khadanga S., Savage P.D., Gaalema D.E. (2022). Enhancing participation in cardiac rehabilitation: Focus on underserved populations. Prog. Cardiovasc. Dis..

[87] Prescott E., Mikkelsen N., Holdgaard A., Eser P., Marcin T., Wilhelm M., Gil C.P., Gonzalez-Juanatey J.R., Moatemri F., Iliou M.C. (2019). Cardiac rehabilitation in the elderly patient in eight rehabilitation units in Western Europe: Baseline data from the EU-CaRE multicentre observational study. Eur. J. Prev. Cardiol..

[88] Ades P.A., Waldmann M.L., McCann W.J., Weaver S.O. (1992). Predictors of cardiac rehabilitation participation in older coronary patients. Arch. Intern. Med..

[89] Ijaz N., Buta B., Xue Q.L., Mohess D.T., Bushan A., Tran H., Batchelor W., deFilippi C.R., Walston J.D., Bandeen-Roche K. (2022). Interventions for Frailty Among Older Adults With Cardiovascular Disease: JACC State-of-the-Art Review. J. Am. Coll. Cardiol..

[90] MacEachern E., Quach J., Giacomantonio N., Theou O., Hillier T., Abel-Adegbite I., Gonzalez-Lara M., Kehler D.S. (2024). Cardiac rehabilitation and frailty: A systematic review and meta-analysis. Eur. J. Prev. Cardiol..

[91] Wongvibulsin S., Habeos E.E., Huynh P.P., Xun H., Shan R., Porosnicu Rodriguez K.A., Wang J., Gandapur Y.K., Osuji N., Shah L.M. (2021). Digital Health Interventions for Cardiac Rehabilitation: Systematic Literature Review. J. Med. Internet Res..

[92] Linn N., Goetzinger C., Regnaux J.P., Schmitz S., Dessenne C., Fagherazzi G., Aguayo G.A. (2021). Digital Health Interventions among People Living with Frailty: A Scoping Review. J. Am. Med. Dir. Assoc..

[93] Strike S.C., Carlisle A., Gibson E.L., Dyall S.C. (2016). A High Omega-3 Fatty Acid Multinutrient Supplement Benefits Cognition and Mobility in Older Women: A Randomized, Double-blind, Placebo-controlled Pilot Study. J. Gerontol. A Biol. Sci. Med. Sci..

[94] Taylor J.S., DeMers S.M., Vig E.K., Borson S. (2012). The disappearing subject: Exclusion of people with cognitive impairment and dementia from geriatrics research. J. Am. Geriatr. Soc..

[95] Makita S., Yasu T., Akashi Y.J., Adachi H., Izawa H., Ishihara S., Iso Y., Ohuchi H., Omiya K., Ohya Y. (2022). JCS/JACR 2021 Guideline on Rehabilitation in Patients With Cardiovascular Disease. Circ. J..

[96] Alosco M.L., Brickman A.M., Spitznagel M.B., Sweet L.H., Josephson R., Griffith E.Y., Narkhede A., Hughes J., Gunstad J. (2015). Daily Physical Activity Is Associated with Subcortical Brain Volume and Cognition in Heart Failure. J. Int. Neuropsychol. Soc..

[97] O’Caoimh R., Gao Y., McGlade C., Healy L., Gallagher P., Timmons S., Molloy D.W. (2012). Comparison of the quick mild cognitive impairment (Qmci) screen and the SMMSE in screening for mild cognitive impairment. Age Ageing.

[98] Patru O., Luca S., Cozma D., Vacarescu C., Crisan S., Valcovici M.D., Virtosu M., Zus A.S., Luca C.T., Dragan S.R. (2025). Cardiac Rehabilitation in the Era of CRT and ARNI: A Missing Link in Heart Failure with Reduced Ejection Fraction Care. J. Clin. Med..

